# Does Continuous Positive Airway Pressure Improve Liver Outcomes in MASLD with Obstructive Sleep Apnea? A Systematic Review

**DOI:** 10.3390/jcm15010225

**Published:** 2025-12-27

**Authors:** Theja V. Channapragada, Clinton R. Brenner, Keven Guruswamy, Rewanth Katamreddy, Alwyn T. Pandian, Vyshnavi Pendala, Jaydon J. Sam, Jonathan G. Stine, Michael J. Brenner, Vinciya Pandian

**Affiliations:** 1 Penn State Health Milton S. Hershey Medical Center, Hershey, PA 17033, USA; tchannapragada@pennstatehealth.psu.edu (T.V.C.); jstine@pennstatehealth.psu.edu (J.G.S.); 2 College of Literature, Science, and the Arts, University of Michigan, Ann Arbor, MI 48109, USA; 3 College of Natural and Mathematical Sciences, University of Maryland Baltimore County, Catonsville, MD 21250, USA; kgurusw1@umbc.edu; 4 Guthrie Medical Center, Sayre, PA 18840, USA; rewanth.katamreddy@guthrie.org; 5 College of Education, The Pennsylvania State University, University Park, PA 16802, USA; atp5521@psu.edu; 6 West Virginia School of Osteopathic Medicine, Lewisburg, WV 24901, USA; vp3sm@virginia.edu; 7 Center for Immersive Learning and Digital Innovation, The Pennsylvania State University, University Park, PA 16802, USA; jjs8788@psu.edu (J.J.S.); vpandian@psu.edu (V.P.); 8 Department of Otolaryngology—Head and Neck Surgery, University of Michigan Medical School, Ann Arbor, MI 48104, USA; 9 Ross and Carol Nese College of Nursing, The Pennsylvania State University, University Park, PA 16802, USA; 10 Department of Otolaryngology—Head and Neck Surgery, College of Medicine, The Pennsylvania State University, Hershey, PA 17033, USA

**Keywords:** obstructive sleep apnea, continuous positive airway pressure, metabolic dysfunction-associated steatotic liver disease, systematic review, OSA, CPAP, MASLD

## Abstract

**Background/Objectives:** Metabolic dysfunction-associated steatotic liver disease (MASLD) often coexists with obstructive sleep apnea (OSA) due to overlapping metabolic risk factors. Whether continuous positive airway pressure (CPAP) influences hepatic outcomes in MASLD remains uncertain. This systematic review, using updated criteria for MASLD, evaluated the effects of OSA treatment on liver and metabolic outcomes. **Methods:** PubMed, Web of Science, and CINAHL were searched for randomized controlled trials (RCTs) and observational studies in adults with MASLD and OSA treated with CPAP, lifestyle interventions, pharmacotherapy, or surgery. Outcomes included liver stiffness, fat content, enzymes, fibrosis scores, HbA1c, lipids, and anthropometrics. Risk of bias was assessed with RoB 2 (RCTs) and ROBINS-I (non-randomized studies) and certainty of evidence with GRADE. **Results:** Eight studies (three RCTs, five observational; n = 1006; 73.5% male) met criteria. Studies evaluated CPAP for from 4 weeks to 3 years, with adherence ≥ 4 h/night in most. CPAP produced modest, inconsistent reductions in alanine aminotransferase and aspartate aminotransferase, small improvements in HbA1c and triglycerides, and minimal changes in liver stiffness, steatosis, weight, or anthropometrics. No RCT demonstrated significant improvement in fibrosis or steatosis. Risk of bias was low in one RCT, “some concerns” in two, and moderate in observational studies; one study had serious confounding risk. **Conclusions:** CPAP may modestly improve liver enzymes and select metabolic parameters in MASLD with OSA, but evidence for salutary effects on steatosis, fibrosis, and body composition is limited. Level of evidence was low due to methodological limitations, heterogeneity, and imprecision. High-quality, longitudinal trials are needed.

## 1. Introduction

Metabolic dysfunction-associated steatotic liver disease (MASLD) affects roughly one in four adults worldwide and is now a leading cause of chronic liver-related morbidity and mortality [1]. Its high burden stems from metabolic risk factors such as obesity, insulin resistance, and dyslipidemia as well as potentially modifiable coexisting conditions. Among these, obstructive sleep apnea (OSA), characterized by recurrent upper airway collapse and chronic intermittent hypoxia (CIH), is emerging as a key, yet underappreciated, contributor to liver injury [2,3].

Clinical and mechanistic studies suggest that CIH amplifies oxidative stress, systemic inflammation, insulin resistance, and adipose lipolysis. These changes accelerate hepatic steatosis, inflammation, and fibrogenesis [2,3]. Hypoxia-inducible signaling impairs hepatic lipid metabolism, and preventing this hypoxia mitigates liver injury, reinforcing the biological plausibility of an OSA–MASLD interaction [4,5]. Randomized controlled trials of continuous positive airway pressure (CPAP) have yielded inconsistent results, reflecting variability in study design, CPAP regimen, or metabolic confounders [6]. However, few studies have applied the 2023 international consensus redefinition of MASLD, which provides a unified pathophysiological framework that parallels mechanisms implicated in OSA [7].

A critical gap persists in understanding whether treating OSA-related hypoxia improves liver health. To address this, we conducted a systematic review, among the first to apply the updated MASLD nomenclature, to evaluate whether CPAP affects hepatic and metabolic endpoints. Our objectives were to (1) identify and appraise studies of OSA-directed therapies (CPAP, lifestyle, hypoglossal nerve stimulation, bariatric surgery); (2) synthesize their effects on hepatic outcomes (e.g., liver stiffness, fat content, aminotransferases, fibrosis scores) and metabolic outcomes (e.g., hemoglobin A1c (HbA1c), lipids, body weight/anthropometrics); and (3) rate certainty and risk of bias using the Cochrane Risk of Bias 2 tool (ROB-2)/the Risk Of Bias In Non-randomized Studies of Interventions tool (ROBINS-I) and the Grading of Recommendations Assessment, Development and Evaluation approach (GRADE) to inform practice and research priorities.

## 2. Materials and Methods

### 2.1. Protocol and Registration

This review followed PRISMA 2020 guidance. The PRISMA 2020 Checklist is attached as Table S1. The protocol was prospectively registered in the International Prospective Register of Systematic Reviews (PROSPERO) under registration number CRD420251128911 (https://www.crd.york.ac.uk/PROSPERO/view/CRD420251128911) (accessed on 19 November 2025).

### 2.2. Eligibility Criteria

We sought original research studies involving adults (≥18 years) diagnosed with MASLD, metabolic dysfunction-associated steatohepatitis (MASH), nonalcoholic fatty liver disease (NAFLD), metabolic dysfunction-associated fatty liver disease (MAFLD), in combination with OSA (Table 1) and treated with CPAP. Randomized controlled trials (RCTs), prospective and retrospective cohort studies, and comparative observational designs were included. Outcomes of interest included measures related to liver health (liver enzymes, liver stiffness, hepatic fat content by imaging, histologic fibrosis scores) and metabolic health (HbA1c, lipid profiles, body weight, and anthropometric measures). Studies involving patients with alcohol-related liver disease, viral hepatitis, central sleep apnea, hepatocellular carcinoma, or end-stage liver disease were excluded. Studies without relevant comparator, no liver/metabolic outcomes, or lacking intervention were also excluded. Reviews, commentaries, conference abstracts, small case series, and non-English articles were also excluded.

### 2.3. Information Sources, Search Strategy, and Selection Process

A comprehensive systematic search was conducted across PubMed, Web of Science, and Cumulative Index to Nursing and Allied Health Literature (CINAHL) from database inception through the final search date in April 2025. No temporal or publication-type restrictions were applied beyond language and population filters. To ensure completeness, we also reviewed reference lists of included studies and relevant reviews; no additional records were identified through citation chaining, clinical trial registries, or grey literature searches. The full search strategies, including controlled vocabulary terms and Boolean operators, are provided in Supplementary File S1. Searches were limited to English-language human studies in adults. All retrieved records were imported into Covidence for de-duplication, screening, and data management. Two reviewers independently screened titles and abstracts, followed by full-text reviews of potentially eligible articles. Discrepancies were resolved through consensus discussion and adjudication by a third reviewer.

### 2.4. Data Collection Process and Extraction

Two reviewers independently extracted data from each included study using Covidence, following a structured protocol to ensure consistency, transparency, and reproducibility. Data collection instruments were developed a priori and iteratively refined after pilot testing to align with the review’s objectives. Custom data sheets were designed to capture information across the Population, Intervention, Comparison, and Outcome (PICO) domains including population, intervention, comparator, and outcomes, in addition to recording methodological and contextual features. Reviewers recorded study identifiers, design type, inclusion and exclusion criteria, and setting, along with details about participant recruitment, analytic timeframe, and quality appraisal domains. To support cross-study comparability, standardized definitions and coding conventions were applied for all extracted fields. Each record underwent independent verification by both reviewers, with discrepancies resolved through discussion or adjudication by a third reviewer. This process ensured uniform data capture and minimized bias in the synthesis phase.

### 2.5. Data Items

Data items included hepatic outcomes such as the liver enzymes alanine aminotransferase (ALT) and aspartate aminotransferase (AST), liver stiffness measured by vibration-controlled transient elastography (VCTE) or magnetic resonance elastography (MRE), hepatic fat content measured by magnetic resonance imaging, controlled attenuation parameters (CAPs) or computed tomography (CT), and histologic or other noninvasive fibrosis scores, alongside metabolic markers (glycated HbA1c, triglycerides, low-density lipoprotein cholesterol [LDL-C], high-density lipoprotein cholesterol [HDL-C]) and anthropometrics/body composition (body mass index [BMI], body weight, waist and neck circumference, waist-to-hip ratio). For studies with multiple time points, outcomes were extracted at baseline and the prespecified follow-up. Additional variables included study design, country, sample size, intervention type and duration, adherence, diagnostic methods for hepatic steatosis and OSA, population characteristics (age, sex, body mass index (BMI), OSA severity), and comorbidities (diabetes, hypertension). Participants were classified having MASLD if they had hepatic steatosis (by imaging or histology) plus ≥ 1 cardiometabolic risk factor (overweight/obesity, type 2 diabetes/, dyslipidemia, hypertension, or increased waist circumference); high-risk alcohol intake was considered metabolic dysfunction and alcohol-related liver disease and excluded. Assumptions were made when categorizing unclear intervention durations or diagnostic methods, which were verified by consensus during data extraction. See Table 1 for eligibility criteria for study selection.

### 2.6. Study Risk of Bias Assessment

Two reviewers independently assessed the risk of bias for each study. RCTs were evaluated using the Cochrane Risk of Bias 2.0 (RoB 2) tool which assess bias in five domains: randomization process, deviation from intended interventions, missing outcome data, measurement of the outcome, and selection of the reported result. Non-randomized studies were assessed using the ROBINS-I tool, which evaluates bias due to confounding, participant selection, classification of interventions, deviations from interventions, deviations from intended interventions, missing data, outcome measurement, and selection of reported results.

Domain judgments were summarized into overall ratings per tool guidance (RoB 2: low, some concerns, high; ROBINS-I: low, moderate, serious, critical, or no information). Disagreements were resolved by consensus/third reviewer. Visual summaries used standard RoB plotting templates. No tool adaptations were carried out, and authors were not contacted for additional risk-of-bias information.

### 2.7. Effect Measures

Due to heterogeneity in study design and outcome reporting, standardized effect measures were not uniformly calculated. Descriptive statistics (mean changes, percentages, and direction of effect) were used to present narrative synthesis of findings. Where available, reported means and standard deviations were extracted to support narrative comparison.

### 2.8. Synthesis Methods

Studies were grouped by intervention; only CPAP studies met inclusion criteria; thus, synthesis focused on CPAP intervention. Data were primarily reported as means with standard deviations, requiring minimal transformation. When summary statistics were incomplete or unavailable, outcomes were described narratively. Data were exported from Covidence into structured Excel tables for organization and comparison. Visual summary tables were created to present study characteristics, participant demographics and comorbidities, CPAP intervention details, and clinical outcomes.

Meta-analysis was not feasible due to heterogeneity in design, populations, diagnostics, and outcomes. A narrative synthesis emphasizing directionality and clinical relevance was conducted. Heterogeneity was described qualitatively (intervention type, diagnostic methods, outcome definitions). No subgroup or sensitivity analyses were performed.

### 2.9. Reporting Bias Assessment

Risk of bias due to missing results was considered during the selection process. We assessed for outcome reporting bias within each included study using ROB tools. No formal tools (e.g., funnel plots) were applied due to the absence of meta-analysis. Study authors were not contacted for unpublished outcomes, and the assessment of reporting bias was limited to information available in published reports.

### 2.10. Certainty Assessment

We assessed the certainty of evidence using the GRADE Handbook [8]. Four reviewers independently rated each outcome across standard domains of study limitations (risk of bias), inconsistency, indirectness, imprecision, and publication bias, with discrepancies resolved through consensus or adjudication by a senior reviewer to ensure methodological rigor and reproducibility. For randomized trials, certainty ratings started high and were downgraded based on identified concerns; for observational studies, ratings started low and could be upgraded for large effects, dose–response gradients, or plausible residual confounding. Risk of bias was assessed using RoB 2 for randomized controlled trials and ROBINS-I for non-randomized studies. All ratings and rationales were documented in evidence profiles, with overall certainty categorized as high, moderate, low, or very low in accordance with GRADE recommendations.

## 3. Results

**Figure 1:**
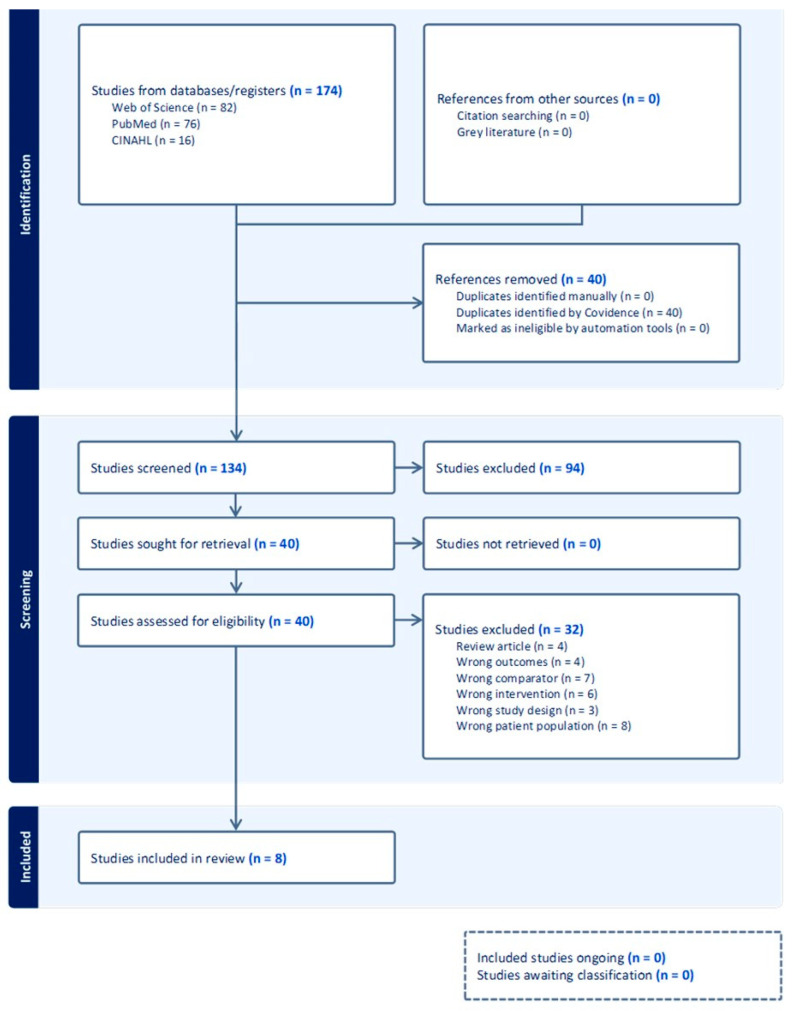
Study flow chart following PRISMA 2020 guidelines.

In this study selection, a total of 174 records were identified through database searches: 82 from Web of Science, 76 from PubMed, and 16 from CINAHL (Figure 1). After removing 40 duplicates via Covidence, 134 records underwent title/abstract screening. Of these, 94 were excluded based on relevance, and 40 full-text articles were assessed for eligibility. Thirty-two studies were excluded for the following reasons: wrong outcomes (n = 4), wrong comparator (n = 7), wrong intervention (n = 4), wrong study design (n = 7), wrong patient population (n = 8), and inadequate comparative data (n = 2). No records were listed as not retrieved. Eight studies met inclusion criteria.

### 3.1. Study and Participant Characteristics

Studies were conducted in Japan, France, Switzerland, the United States, the United Kingdom, Hong Kong, China, and Israel (Table 1). Study designs included three RCTs, two prospective cohorts, two retrospective cohorts, and one additional observational cohort (Table 2). Sample sizes ranged from 47 to 351 participants. Most enrolled adults with moderate-to-severe OSA (apnea–hypopnea index [AHI] ≥ 15 events/h) and excluded significant liver disease, high alcohol intake, or prior OSA therapy. OSA was diagnosed primarily by polysomnography (PSG); however, some studies used the respiratory event index (REI) or oxygen desaturation index (ODI). Diagnosis of hepatic steatosis was performed through ultrasound, VCTE (CAP), CT attenuation, liver enzymes, or biopsy Magnetic Resonance Spectroscopy (MRS) with intrahepatic triglyceride content (IHTG) ≥ 5%. Six studies confirmed hepatic steatosis by imaging; two were biomarker-only (enzymes or FibroTest panels).

A total of 1006 patients were included across the eight studies (Table 2). The population was predominantly male (739; 73.5%). Mean participant ages ranged from 42.6 to 57.6 years, with BMI ranging between 26.28 and 35.00 kg/m^2^. OSA severity distributions varied by study with severe OSA predominating in Shpirer et al. and Chen et al. including a broader AHI range [15,16].

Comorbidities such as diabetes and hypertension were inconsistently reported across studies. The prevalence of diabetes ranged from 0% to 43.8% among controls and 2.1% to 43.8% in intervention groups, with higher prevalence rates noted in Shpirer et al. and Kim et al. [15,16]. Hypertension prevalence ranged from 21.3% to 81.3%. Several studies did not comprehensively report comorbidity data [6,9,14] (see Table 3).

### 3.2. Interventions

Interventions consisted exclusively of CPAP; reported mean nightly use generally met the ≥4 h/night benchmark [8,9,10,11,12,13,14] (Table 4). Three RCTs used sham or sub-therapeutic CPAP comparators; two cohort studies had internal comparators, and the remainder were pre–post single-arm (Table 4). Duration of therapy ranged from 4 weeks to 3 years, with most studies reporting interventions lasting at least 3 to 6 months. Adherence levels were generally moderate to high, with average use ranging from 3.1 ± 2.4 h to 4.8 ± 2.1 h [8,9,10,11,12,13,14]. Across included studies, CPAP adherence met or exceeded the clinical benchmark of ≥4 h per night; Shpirer et al. reported ≥5 h, and Kohler et al. noted 4.6 ± 2.1 h [10,15]. Kim et al. defined compliance as >4 h/night or >70% usage over ≥3 months [13]. Hirono et al. used a modified compliance index (m-CI > 0.5) which was a surrogate for good adherence [9].

### 3.3. Clinical Outcomes

CPAP therapy had variable effects on hepatic, metabolic, and anthropometric outcomes (Table 5a,b). In terms of hepatic outcomes, liver stiffness remained largely unchanged, with minor variations reported in Hirono et al. and Ng et al. [6,9]. Kim et al. reported improved AST-to-Platelet Ratio Index (APRI) scores, and Jullian-Desayes et al. reported favorable shifts in the FibroTest/SteatoTest serologic scores [11]. Liver fat content changes were negligible across studies. ALT and AST generally trended downward with CPAP, most consistently in adherent subgroups and longer follow-up.

With regards to metabolic outcomes, CPAP therapy was associated with modest improvements in HbA1c and triglycerides, though HDL and LDL levels remained stable [9,15]. In terms of anthropometrics, most studies showed minimal change in BMI or waist circumference; however, these were only reported for four studies. Toyama et al. observed negligible changes in BMI and waist circumference, while Hirono et al. noted slight weight loss in participants [10,14].

### 3.4. Risk of Bias

**Figure 2:**
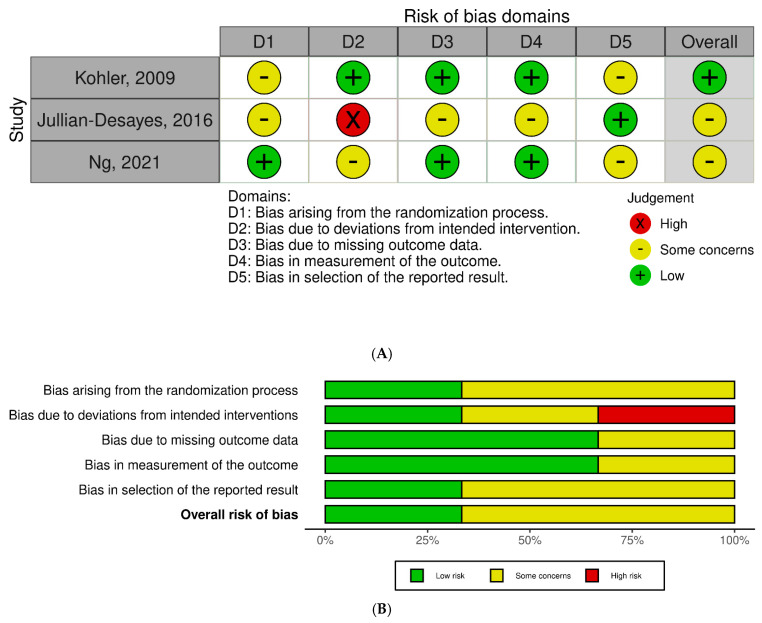
(**A**) Risk of bias assessment for randomized controlled trials using the Cochrane RoB 2 tool [6,10,11]. (**B**) Summary of overall risk of bias judgments for randomized controlled trials.

**Figure 3:**
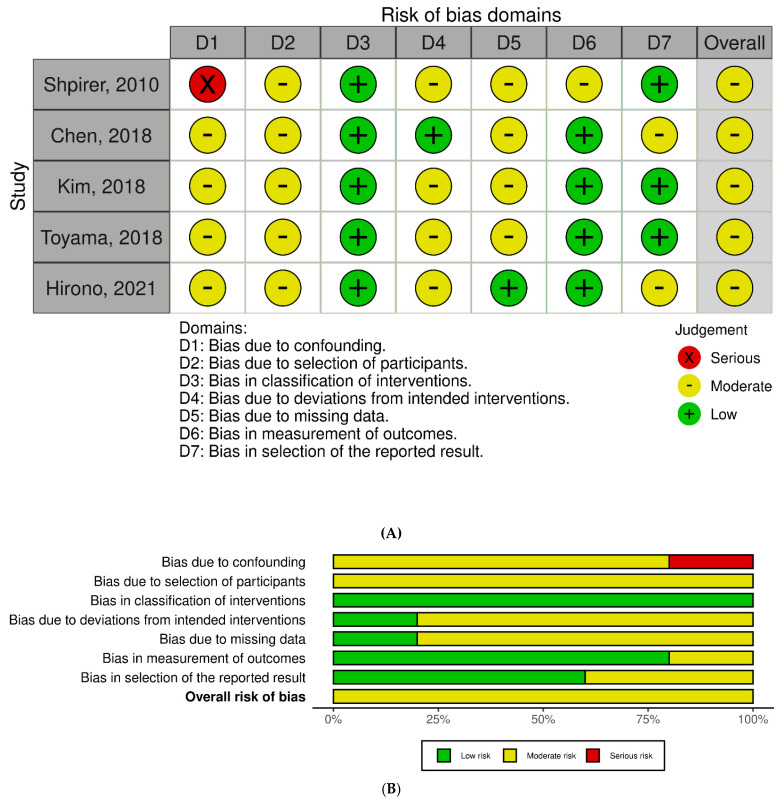
(**A**) Risk of bias assessment for non-randomized studies using ROBINS-I tool [9,13,14,15,16]. (**B**) Summary distribution of ROBINS-I risk of bias judgments across non-randomized studies.

Among RCTs, overall judgments ranged from low to some concerns. Kohler et al. was largely low risk with concerns in the randomization process and outcome measurement; Jullian-Desayes et al. and Ng et al. were judged as having some concerns due to deviations from intended interventions and issues in randomization/allocation or outcome measurement (Figure 2A,B) [6,10,11]. For non-randomized studies (ROBINS-I), overall risk was predominantly moderate, driven by confounding and selection of participants; Shpirer et al. had serious confounding risk (Figure 3A,B). Results should be interpreted cautiously given design limitations and potential residual confounding.

### 3.5. Reporting Biases and Uncertainty

Among the excluded studies were two studies that evaluated bariatric surgery. However, their outcome measures were unique and not comparable to the CPAP-focused studies and thus were not reported. No evidence of selective reporting bias was identified, but formal statistical assessment (funnel plots, Egger’s test) was not feasible without meta-analysis. GRADE assessments revealed that the certainty of evidence was generally low to very low across outcomes, largely due to methodological limitations, heterogeneity, and imprecision (Table 6).

## 4. Discussion

In this systematic review of adults with OSA and MASLD, positive airway pressure produced modest improvements in aminotransferases without changes in imaging-based steatosis or stiffness. The largest randomized trial estimating intrahepatic triglyceride content by MRS with CPAP compliance (≈4.4 h/night) found no reduction in liver fat at six months [6]. Furthermore, pooled trials comparing CPAP with sham did not improve composite fibrosis/steatosis indices [11]. Across included prospective cohorts and randomized trials, small declines in ALT/AST were observed, and greater improvements in ALT/AST were reported among participants with increased duration of CPAP beyond three months of therapy [9,12,13,14,15]. The pattern of biochemical change without concomitant reductions in fat or stiffness may stem from CPAP potentially attenuating hypoxia-driven systemic inflammation and insulin resistance rather than the induction of short-term structural remodeling of the liver [2].

This study is one of the first to utilize the 2023 MASLD framework in the context of OSA, requiring objective hepatic steatosis plus ≥1 cardiometabolic risk factors (e.g., overweight/obesity, type 2 diabetes, dyslipidemia, hypertension, or other metabolic dysregulation) and excluding secondary causes [7]. Substantial heterogeneity in study designs to quantify hepatic steatosis as well other hepatic/metabolic parameters made comparability across studies limited. All studies in this review utilized legacy terminology of NAFLD, MAFLD, and fatty liver, and diagnosis of hepatic steatosis ranged from ultrasound and CAP (VCTE) to non-contrast CT, biopsy, and MRS.

### 4.1. Heterogeneity in OSA and Hepatic Assessment

While liver biopsy is the gold standard test, magnetic resonance imaging–proton density fat fraction (MRI-PDFF) is the noninvasive reference standard for quantifying hepatic steatosis and provides an accurate, reproducible, and precise measurement of liver fat content with relative independence variability from BMI and fibrosis [17]. MRS is an acceptable high-precision alternative when available. CAP is accessible and scalable but less accurate than MR-based methods and is influenced by BMI as well [18]. CT can detect moderate to severe steatosis but lacks sensitivity for mild disease, does not reliably quantify longitudinal change, and exposes patients to ionizing radiation [18]. No studies used MRI-PDFF. Liver biopsy remains the reference standard for staging fibrosis but is invasive. Among noninvasive options, MRE offers the highest accuracy, while VCTE is the preferred first-line tool for screening and risk stratification [17].

Heterogeneity also impacted OSA phenotyping. While many studies used PSG with AHI, which is considered the gold standard for diagnosing OSA, others relied on home testing with the REI, which is calculated per hour of recording rather than sleep and can underestimate severity [19]. ODI definitions and cutoffs varied, further complicating cross-study severity matching and adherence response analyses [20]. Adherence definitions were non-standard, ranging from hours per night vs. the proportion of nights with ≥4 h of use. Moreover, follow-up was typically short, less than 6 months in most cohorts. These timelines are ill-suited to detect changes in imaging-based steatosis or fibrosis; while steatosis is dynamic and can rise or fall within weeks, fibrosis accrues slowly over years [21].

### 4.2. Serum-Based Indices and Composite Panels

Two studies in our review reported changes in serum-based indices. The APRI, a simple score derived from AST and platelet count intended to estimate fibrosis, showed improvement in more adherent CPAP users [13]. APRI is heavily driven by aminotransferase levels and performs less well than the fibrosis-4 index (FIB-4) and nonalcoholic fatty liver disease fibrosis score (NFS) in MASLD, with reductions likely reflecting biochemical fluctuation rather than true fibrosis regression over short follow-up period [22]. Similarly, FibroTest/SteatoTest, proprietary multi-analyte panels that estimate fibrosis and steatosis, shifted favorably in Jullian-Desayes et al. [11]. Only direction of change was reported, however, with no baseline/follow-up values or confidence intervals, limiting interpretability and precluding synthesis.

### 4.3. Quality of Evidence and Gaps

This methodological diversity prevented a meta-analysis of hepatic endpoints, despite broad database searches and independent dual screening and extraction using RoB 2 for randomized trials and ROBINS-I for non-randomized studies. This heterogeneity, along with small sample sizes, focused this review as a narrative approach centered on direction and consistency of effects rather than pooled estimates. Risk-of-bias judgments further tempered certainty. Among randomized trials, overall assessments ranged from low risk to “some concerns” largely due to issues in randomization, outcome measurement, confounding, and participant selection. We found no evidence of selective reporting among included studies, but formal small-study assessments (funnel plots, Egger’s test) were not feasible without meta-analysis. Using GRADE, certainty of evidence for hepatic outcomes was generally low to very low due to risk of bias, inconsistency, indirectness of measurement, and imprecision.

The absence of studies combining CPAP with lifestyle intervention or pharmacotherapy represents a significant evidence gap, especially given that MASLD has a multifactorial pathogenesis. None of our studies tested the synergistic effect of structured lifestyle programs, specifically prespecified diet and/or aerobic/anabolic exercise regimens with CPAP. Two studies evaluating bariatric surgery were excluded because their outcome measures were not comparable with those used in the CPAP-focused literature, precluding synthesis. This gap limits guidance on multimodal strategies that may deliver greater hepatic benefit synergistically than CPAP alone.

This review integrates evidence across imaging-based and biomarker outcomes, advancing work from earlier studies. A meta-analysis by Chen found small yet significant ALT/AST reductions with CPAP particularly with longer treatment but did not evaluate imaging endpoints [16]. Our data suggest that biochemical improvements rarely translate into reduced liver fat or stiffness within 6 months; studies utilizing MRI-PDFF to quantify hepatic steatosis and VCTE and/or MRE are needed to assess fibrosis [16]. The RCT-only meta-analysis by Labarca et al. had insufficient data to recommend CPAP for nonalcoholic steatohepatitis (NASH). Our MASLD-framed, mixed-design review (RCTs plus prospective cohorts) supports use of CPAP for sleep and cardiometabolic benefits, although lack of evidence for short-term hepatic remodeling suggests a need for multimodal metabolic therapy or longer follow-up [23]. Exclusion of non-English studies is a limitation of the review process.

### 4.4. Future Research Priorities

Future research should prioritize standardization, adequate duration, and comparative effectiveness. For OSA phenotyping, polysomnography-based AHI should anchor eligibility and endpoints, with prespecified supplemental indices utilized (e.g., ODI, hypoxic burden) for uniformity. For hepatic outcomes, MRI-PDFF should be the reference method to diagnose and quantify steatosis, complemented by standardized VCTE or MRE-derived liver stiffness when fibrosis is a primary endpoint. Adherence should be captured and reported with predefined thresholds (e.g., ≥4 h/night on ≥70% of nights), alongside residual respiratory event rates. Adherence-adjusted analyses should be performed. Trials should extend to at least 12 months when imaging or fibrosis outcomes are primary and should directly compare CPAP alone with CPAP plus effective metabolic therapy (e.g., glucagon-like peptide-1 receptor agonists), structured lifestyle programs, and bariatric surgery, with combination arms designed to test the synergistic effects of interventions compared to controls.

## 5. Conclusions

In adults with MASLD and OSA, CPAP improves biochemical and metabolic markers but has not shown consistent effects on liver fat or fibrosis, based on short-term follow-up. The current standard of care for OSA supports CPAP therapy for sleep and cardiometabolic benefits, and further data are needed to determine long-term efficacy in preventing progression of liver disease. Importantly, most included studies achieved only partial nocturnal CPAP exposure (typically defined as ≥4 h/night), and current findings do not establish the hepatic benefits at the adherence levels studied. The current literature represents an absence of high-certainty evidence rather than evidence of an absence of therapeutic efficacy. The lack of structural hepatic change over shorter follow-up intervals may reflect study duration, outcome assessment, heterogeneity, or uncertain therapeutic potential. Future prospective standardized trials are needed to clarify CPAP’s long-term effects on liver health.

## Figures and Tables

**Table 1:** Eligibility Criteria for Study Selection.

Domain	Inclusion Criteria	Exclusion Criteria
Population	Adults ≥ 18 years Diagnosed with the following: ○ MASLD ○ MASH ○ NAFLD ○ OSA	Diagnosed with: ○ Alcohol-related liver disease, viral hepatitis, or other non-MASLD liver diseases ○ End-stage liver disease, transplant, or HCC ○ Central sleep apnea
Intervention/Exposure	CPAP Lifestyle changes (diet, exercise) GLP-1/GIP agents (e.g., Ozempic, Wegovy) Hypoglossal nerve stimulators Bariatric surgery	No CPAP use in CPAP studies Unrelated interventions Non-standard lifestyle regimens Drugs outside metabolic class (e.g., statins, metformin)
Comparator	Any relevant comparator	No comparator or control group
Outcomes	At least one of the following: ○ ≥30% reduction in MRI-PDFF liver fat ○ ≥25% reduction in VCTE or ≥15% in MRE stiffness ○ ALT drop ≥ 17 IU/L or change in AST/ELF ○ ≥5% body weight loss ○ Improved lipids, glycemia, BP, body composition ○ HRQOL or other patient-reported outcomes	No liver fat/stiffness data No liver enzymes or metabolic outcomes No weight loss or HRQOL data
Study Characteristics	Original research: RCTs, cohorts, or comparative observational studies Peer-reviewed	Reviews, commentaries, case reports < 10 patients Non-English Insufficient data or unclear methods Abstract-only
Language and Sample Size	Full text in English Human studies	Animal or in vitro studies

MASLD = metabolic dysfunction-associated steatotic liver disease; MASH = metabolic dysfunction-associated steatohepatitis; NAFLD = nonalcoholic fatty liver disease; OSA = obstructive sleep apnea; CPAP = continuous positive airway pressure; GLP-1 = glucagon-like peptide-1; GIP = gastric inhibitory polypeptide; MRI-PDFF = magnetic resonance imaging–proton density fat fraction; VCTE = vibration-controlled transient elastography; MRE = magnetic resonance elastography; ALT = alanine aminotransferase; AST = aspartate aminotransferase; ELF = enhanced liver fibrosis; HRQOL = health-related quality of life; RCT = randomized controlled trial; HCC = hepatocellular carcinoma; BP = blood pressure.

**Table 2:** Summary of Study Characteristics Investigating MASLD and OSA.

Author (Year)	Country	Design	Population	N	Method of Diagnosis of Steatohepatitis	OSA Diagnosis
Hirono, 2021 [9]	Japan	Prospective cohort	Adults with OSA and NAFLD	70 Total: 20 non-NAFLD (OSA) and 50 NAFLD (CPAP pre–post)	US + FibroScan (LSM, CAP)	PSG; apnea–hypopnea index (AHI) > 15 events/h
Ng, 2021 [6]	Hong Kong	RCT	Adults with OSA and NAFLD	120 total 60 sub-therapeutic CPAP (4 cm H_2_O) 60 auto-CPAP (4–20 cm H_2_O)	Hepatic fat by ^1^H-MRS (IHTG %); FibroScan CAP/LSM as secondary	REI > 5 events/h
Kohler, 2009 [10]	UK	RCT	Adults with OSA	94 total	Liver enzymes as markers, no imaging performed	PSG; ODI ≥ 10
Jullian-Desayes, 2016 [11]	France, Switzerland	RCT	Adults with moderate-to-severe OSA and suspected NAFLD (per FibroMax)	103 total 51CPAP 52 sham	FibroMax (SteatoTest, NashTest, FibroTest)	PSG; AHI > 15 events/h
Chen, 2017 [12]	China	Observational cohort (cross-sectional + pre–post in OSA subset)	Adults undergoing standard polysomnography; OSA subgroup evaluated before/after CPAP	160 total (controls 30; moderate OSA 42; severe OSA 88). CPAP subset: 28 (pre–post, single-arm)	Ultrasound steatosis (grade 0–3) by standard sonographic criteria ARFI elastography (m/s) for fibrosis	PSG (AHI categories: <5 (none), 5–30 (moderate), ≥30 events/h (severe)
Kim, 2018 [13]	USA	Retrospective cohort	Adults with OSA	351 (single-arm)	ALT thresholds; APRI for fibrosis	PSG; AHI used for diagnosis (≥5 events/h) and severity (mild 5–14, moderate 15–29, severe ≥ 30)
Toyama, 2018 [14]	Japan	Retrospective cohort	Adult men with OSA and obesity; CT-defined fatty liver	61 (single-arm); baseline 25 FL/36 non-FL; follow-up after ~31 months	Non-contrast CT; fatty liver = liver CT value ≤ 50 HU (~≥15% steatosis)	PSG; AHI used for diagnosis/severity
Shpirer, 2010 [15]	Israel	Prospective cohort	Adults with OSA and CT-defined fatty liver at baseline	47 total	Non-contrast CT liver attenuation index (LAI); “low LAI” ≤ −10 ≈ ≥ 30% macrovesicular steatosis	AHI > 5+

AHI = apnea–hypopnea index; ALT = alanine aminotransferase; APRI = AST-to-Platelet Ratio Index; CAP = controlled attenuation parameter; CPAP = continuous positive airway pressure; CT = computed tomography; HU = Hounsfield units; IHTG = intrahepatic triglyceride content; LAI = liver attenuation index; LSM = liver stiffness measurement; MRS = Magnetic Resonance Spectroscopy; NAFLD = Nonalcoholic fatty liver disease; OSA = obstructive sleep apnea; ODI = oxygen desaturation index; PSG = polysomnography; RCT = randomized controlled trial; REI = respiratory event index; US = ultrasound.

**Table 3:** Population Characteristics and Outcome Measures Across Included Studies.

Author, Year	Age (mean ± SD)		BMI kg/m^2^ (mean ± SD)		OSA Severity		Diabetes		Hypertension	
	Control	Intervention	Control	Intervention	Control	Intervention	Control	Intervention	Control	Intervention
Hirono, 2021 [9]	54.7 ± 14.1	-	27.6 ± 0.6	27.4 ± 4.4	AHI > 15 h^−1^	AHI > 15 h^−1^	12%	-	40%	-
Ng, 2021 [6]	55 ± 15	55 ± 10	28 ± 4.6	27.5 ± 5.4	REI: 27.9 ± 28.0	REI: 22.5 ± 30.3	-	-	-	-
Kim, 2018 [13]	Mild OSA: 54.2 ± 16.5 Moderate OSA: 57.7 ± 15.0 Severe OSA: 59.0 ± 13.8	-	Mild OSA: 31.6 ± 10.1 Moderate OSA: 29.9 ± 6.4 Severe OSA: 33.9 ± 9.3	32.2 ± 8.0	Mild OSA: AHI 9.8 ± 3.1 ODI 4.1 ± 3.3 Moderate OSA: AHI 21.3 ± 4.3 ODI 11.0 ± 6.1 Severe OSA: AHI 57.8 ± 23.6 ODI 42.2 ± 27.9	AHI: 37.2 ±27.0 ODI: 24.5 ± 26.2	-	30.8%	-	-
Toyama, 2018 [14]	57.2 ± 12.0	59.9 ± 12.1	28.6 ± 4.7	28.7 ± 4.4	AHI: 46.1 ± 18.7, ODI: 33.4 ± 20.5	ODI: 3.4 ± 1.9	-	-	-	-
Chen, 2017 [16]	42.2 ± 12.0	-	24.9–29.5	28.0 ± 4.4	AHI: 2.0–60.1	3.8 ± 3.0	0–5%	-	5–24%	-
Jullian-Desayes, 2016 [11]	57	57	28.5	28.1	AHI: 31.3 h^−1^	AHI: 42.8 h^−1^	3.8%	7.8%	28.8%	25.5%
Shpirer, 2010 [15]	55.7 ± 8.4	52.8 ± 6.8	31.3 ± 4.1	36.1 ± 6.2	Mild: 19.4% Moderate: 29% Severe: 51.6%	Mild: 0 Moderate: 6.3% Severe: 93.8%	25.8%	43.8%	61.3%	81.3%
Kohler, 2009 [10]	48.5 ± 10.4	48.0 ± 9.5	34.4 ± 4.6	35.6 ± 7.1	-	-	0%	2.1%	25.5%	21.3%

Abbreviations: SD = standard deviation; OSA = obstructive sleep apnea; AHI = apnea–hypopnea Index; ODI = oxygen desaturation index.

**Table 4:** Interventions.

Author, Year	CPAP Therapy Duration	Description of Intervention	
**Randomized Controlled Studies**		**Control Group**	**Intervention Group**
Kohler, 2009 [10]	4 weeks	Subtherapeutic CPAP	Titrated CPAP
Ng, 2021 [6]	6 months	Subtherapeutic CPAP	Auto-titrating CPAP
Jullian-Desayes, 2016 [11]	6–12 weeks	Sham CPAP	Auto-titrating CPAP
**Pre-Post Intervention Studies**		**Pre-Intervention**	**Post-Intervention**
Hirono, 2021 [9]	6 months	No CPAP	CPAP
Kim, 2018 [13]	6 months	No CPAP	CPAP
Toyama, 2018 [14]	31.3 ± 6.2 months	No CPAP	CPAP
Chen, 2017 [16]	3 months	No CPAP	CPAP
Shpirer, 2010 [15]	3 years	No CPAP	CPAP

CPAP: continuous positive airway pressure.

**Table 5:** (**a**): Summary of CPAP Impact on Hepatic Outcomes; (**b**): Summary of CPAP Impact on Metabolic and Anthropometric Outcomes.

(**a**)					
**Autdor, Year**	**Hepatic Outcomes**				
	**Liver Stiffness** **Measurement**	**Fibrosis Markers**	**Liver Fat Change**	**Alanine Aminotransferase**	**Aspartate Aminotransferase**
Hirono, 2021 [9]	Pre: 4.6 ± 1.8 kPa Post: 4.7 ± 1.9 kPa [*p* = 0.62]		CAP: Pre: 304.0 ± 52.2 dB/m Post: 303.9 ± 44.1 dB/m [*p* = 0.99]	Pre: 37.6 ± 19.1 Post: 33.1 ± 22.4 [*p* = 0.02]	Pre: 28.2 ± 10.9 Post: 24.7 ± 7.5 [*p* = 0.005]
Ng, 2021 [6]	Auto CPAP: Pre: 6.0 (3.0) kPa Post: 6.2 (2.8) kPa Sub CPAP: Pre: 6.1 (3.4) kPa Post: 6.1 (2.8) kPa [*p* = 0.55]		^1^H-MRS Auto CPAP: Pre: 13.2 ± 7.5% Post: 14.0 ± 9.2% Sub CPAP: Pre: 13.5 ± 9.0% Post: 14.2 ± 8.3% [*p* = 0.97] CAP: Auto CPAP: Pre: 320.4 ± 45.6 dB/m Post: 315.8 ± 43.0 dB/m Sub CPAP: Pre: 316.8 ± 41.7 dB/m Post: 306.9 ± 43.9 dB/m [*p* = 0.42]	Auto CPAP: Pre: 9.2 Post: 6.4 Sub CPAP: Pre: 9.8 Post: 7.2	
Chen, 2017 [16]				Pre: 54.2 ± 24.3 Post: 46.5 ± 25.0 [*p* < 0.001]	Pre: 31.8 ± 8.9 Post: 29.0 ± 8.3 [*p* = 0.04]
Kim, 2018 [13]		APRI: Pre: 0.4 ± 0.3 Post: 0.3 ± 0.2 [*p* = *0*.004]		Pre: 44.5 ± 22.3 Post: 39.1 ± 16.1 [*p* < *0*.001]	Pre: 27.9 ± 15.9 Post: 24.9 ± 9.7 [*p* < *0*.001]
Toyama, 2018 [14]			CT liver value Pre: 38.7 ± 9.2 HU Post: 43.8 ± 10.7 HU [*p* = 0.03]	Pre: 46.0 ± 27.3 Post: 34.6 ± 20.7 [*p* = 0.05]	Pre: 33.9 ± 17.8 Post: 29.8 ± 13.0 [*p* = 0.029]
Jullian-Desayes, 2016 [11]		SteatoTest: no difference FibroTest: no difference NashTest: no difference			
Shpirer, 2010 [15]			Compliant: Pre: −16.7 ± 4.9 HU, LAI Post: 0.4 ± 9.8 HU, LAI [*p* = 0.006] Non-compliant: Pre: −17.3 ± 5.9 HU, LAI Post: −6.5 ± 9.9 HU, LAI [*p* = 0.26]	Compliant: Pre: 35.1 ± 12.6 Post: 24.9 ± 7.8 [*p* = 0.016] Non-compliant: Pre: 29.0 ± 12.8 Post: 41.8 ± 26.2 [*p* = 0.30]	Compliant: Pre: 23.7 ± 5.4 Post: 19.0 ± 2.2 [*p* = 0.024] Non-compliant: Pre: 22.5 ± 8.2 Post: 36.3 ± 19.4 [*p* = 0.16]
Kohler, 2009 [10]				Therapeutic: Pre: 39.1 ± 26.3 Post: 30.3 ± 16.4 Sub-therapeutic: Pre: 36.9 ± 20.7 Post: 31.5 ± 16.5	Therapeutic: Pre: 29.1 ± 14.7 Post: 30.2 ± 13.6 Sub-therapeutic: Pre: 28.2 ± 16.2 Post: 29.5 ± 12.6
(**b**)					
	**Metabolic Outcomes**		**Anthropometric Outcomes**		
	**Glycemic Control (HbA1c)**	**Lipid Profile, mg/dL**	**Weight (BMI kg/m^2^)**		**Body Composition**
Hirono, 2021 [9]	Pre: 6.16 ± 0.78% Post: 6.06 ± 0.55% [*p* = 0.12]	Triglycerides: Pre: 139.5 ± 67.8 Post: 126.0 ± 54.8 [*p* = 0.11] LDL: Pre: 133.9 ± 35.9 Post: 134.2 ± 45.7 [*p* = 0.95] HDL: Pre: 55.9 ± 12.9 Post: 56.1 ± 11.4 [*p* = 0.87]	Pre: 27.6 ± 4.4 Post: 27.4 ± 4.4 kg/m^2^ [*p* = 0.15]		
Ng, 2021 [6]					Pre: 77–79.7 Post: 76.9–79.3 [*p* = 0.17]
Chen, 2017 [16]			Pre: 30.87 (5.51) Post: 30.49 (5.13) [*p* = 0.13]		
Toyoma, 2018 [14]		Triglycerides: Pre: 171.4 ± 70.0 Post: 178.7 ± 137.6 [*p* = 0.763]	Pre: 31.2 ± 5.4 Post: 30.8 ± 4.9 [*p* = 0.27]		Waist circumference: Pre: 100.5 ± 11.6 Post: 99.6 ± 12.0 [*p* = 0.383] Visceral fat area, cm^2^ Pre: 171.0 ± 61.3 Post: 161.9 ± 79.0 [*p* = 0.308]
Shpirer, 2010 [15]		Compliant: Triglycerides: Pre: 222.9 ± 90.3 Post: 194.6 ± 128 [*p* = 0.32] Cholesterol: Pre: 177.4 ± 28.7 Post: 198.4 ± 31.3 [*p* = 0.14] Non-compliant: Triglycerides: Pre: 203.5 ± 93.3 Post: 199.3 ± 22.1 [*p* = 0.91] Cholesterol: Pre: 163.0 ± 71.4 Post: 135.5 ± 19.3 [*p* = 0.49]	Compliant: Pre: 34.8 ± 5.0 Post: 35.0 ± 5.2 [*p* = 0.70] Non-compliant: Pre: 34.1 ± 7.8 Post: 34.9 ± 8.4 [*p* = 0.56]		

*p*-values reflect within-group pre–post comparisons unless otherwise specified. For randomized controlled trials with comparator arms, *p*-values correspond to between-group comparisons as reported in the original studies. CPAP: continuous positive airway pressure; CAP: controlled attenuation parameter; ^1^H-MRS (Proton Magnetic Resonance Spectroscopy); APRI: aspartate aminotransferase-to-Platelet Ratio Index; CT: computed tomography; HUs: Hounsfield units (HU); LAI: liver attenuation index; HbA1c: hemoglobin A1c; BMI: body mass index; LDL: low-density lipoprotein.

**Table 6:** GRADE Summary of Findings: GRADE summary of findings for CPAP in adults with coexisting MASLD and OSA. Across RCTs and non-RCTs, certainty was low for liver enzymes, fibrosis scores, glycemic control, and lipids and very low for liver fat content and anthropometric/body-composition outcomes, primarily due to risk of bias, inconsistency, and imprecision.

Outcome	No. of Studies	Study Design	Risk of Bias	Inconsistency	Indirectness	Imprecision	Publication Bias	Certainty of Evidence
Liver stiffness	3	RCTs and non-RCTs	Moderate to Serious	Serious	Not serious	Serious	Undetected	Low
Liver enzymes (ALT, AST)	6	RCTs and non-RCTs	Moderate	Moderate	Not serious	Moderate	Undetected	Low
Liver fat content (CAP, MRI, IHTG)	4	RCTs and non-RCTs	Moderate	Serious	Not serious	Serious	Undetected	Very Low
Fibrosis score (FibroTest, APRI, ELF)	3	RCTs and non-RCTs	Moderate	Moderate	Not serious	Moderate	Undetected	Low
Glycemic control (HbA1c)	3	RCTs and non-RCTs	Moderate	Moderate	Not serious	Moderate	Undetected	Low
Lipid profile (TG, HDL, LDL)	4	RCTs and non-RCTs	Moderate	Moderate	Not serious	Moderate	Undetected	Low
Anthropometrics (BMI, waist circumference, body weight)	5	RCTs and non-RCTs	Moderate	Serious	Not serious	Serious	Undetected	Very Low
Body composition (waist-to-hip ratio, neck circumference)	2	RCTs and non-RCTs	Moderate	Moderate	Not serious	Serious	Undetected	Very Low

ALT: alanine aminotransferase; AST: aspartate aminotransferase; CAP: controlled attenuation parameter; MRI: magnetic resonance imaging; IHTG: intrahepatic triglyceride content; APRI: aspartate aminotransferase-to-Platelet Ratio Index; ELF: enhanced liver fibrosis (score); HbA1c: hemoglobin A1c; TG: triglycerides; HDL: high-density lipoprotein (cholesterol); LDL: low-density lipoprotein (cholesterol); BMI: body mass index; RCT: randomized controlled trial.

## Data Availability

All data supporting the findings of this study are available within the article and its Supplementary Materials. The datasets generated and analyzed during the current review consist of published studies that are publicly available through the cited sources. No new data were created or analyzed in this study. The protocol was prospectively registered in the International Prospective Register of Systematic Reviews (PROSPERO) under registration number CRD420251128911.

## Supplementary Materials

The following supporting information can be downloaded at: https://www.mdpi.com/article/10.3390/jcm15010225/s1, Table S1: PRISMA 2020 checklist [24]; File S1: The full search strategies.
