# Supplementary material for: Does Continuous Positive Airway Pressure Improve Liver Outcomes in MASLD with Obstructive Sleep Apnea? A Systematic Review
Source: J Clin Med. 2025 Dec 27;15(1):225. doi: 10.3390/jcm15010225 (PMC12787049; doi:10.3390/jcm15010225)
Supplement: Supplementary file 1 [file jcm-15-00225-s001.zip › Supplemental File 1.pdf]

Supplemental File 1

Database	Search String	Outcome
PubMed	(((("Non-alcoholic Fatty Liver Disease"[Mesh]) OR (metabolic dysfunction-associated steatotic liver disease OR MASLD OR nonalcoholic fatty liver disease OR NAFLD OR steatotic liver disease OR nonalcoholic steatohepatitis OR NASH)) AND ((((("Sleep Apnea Syndromes"[Mesh] OR "Sleep Apnea, Obstructive"[Mesh]) OR "Apnea"[Mesh]) OR "Airway Obstruction"[Mesh]) OR "Polysomnography"[Mesh]) OR (obstructive sleep apnea OR OSA OR sleep apnea syndromes OR airway obstruction OR apnea OR polysomnography OR PSG OR sleep disordered breathing OR apneic OR hypopnea OR upper airway resistance syndrome OR UARS OR Apnea Hypopnea Index OR AHI OR Epworth Sleepiness Scale OR ESS OR Oxygen desaturation index OR ODI OR Pittsburgh Sleep Quality Index OR PSQI OR Functional Outcomes of Sleep Questionnaire OR FOSQ OR Sleep efficiency OR Rosen Criteria))) AND ((((((((("Continuous Positive Airway Pressure"[Mesh]) OR "Drug Therapy"[Mesh]) OR "Glucagon-Like Peptide 1"[Mesh]) OR "semaglutide" [Supplementary Concept]) OR "Liraglutide"[Mesh]) OR "Bariatric Surgery"[Mesh]) OR "Tirzepatide"[Mesh]) OR "retatrutide" [Supplementary Concept]) OR (continuous positive airway pressure OR CPAP OR lifestyle modification	76

	OR pharmacological treatment OR pharmacological intervention OR Glucagon-like peptide-1 OR GPL-1 OR semaglutide OR Wegovy OR Ozempic OR liraglutide OR Saxenda OR Victoza OR Hypoglossal nerve stimulator OR surgical intervention OR bariatric surgery OR Tirzepatide (Mounjaro) OR CagriSema OR Retatrutide OR life style changes OR drug therapy OR Pharmacological and Biological Treatments))) AND ((((((("Weight Loss"[Mesh]) OR "Glycemic Control"[Mesh]) OR "Blood Pressure"[Mesh]) OR "Body Composition"[Mesh]) OR "Alanine Transaminase"[Mesh]) OR "Aspartate Aminotransferases"[Mesh]) OR "Elasticity Imaging Techniques"[Mesh]) OR (liver fat OR liver stiffness OR circulating biomarker OR body weight loss OR metabolic disease control OR lipid change OR glycemic control OR blood pressure OR composition OR body composition OR health-related quality of life OR Alanine Transaminase OR HRQOL OR alanine aminotransferase OR ALT OR aspartate aminotransferase OR AST OR enhanced liver fibrosis OR ELF OR magnetic resonance elastography OR MRE OR magnetic resonance imaging proton density fat fraction OR MRI-PDFF OR vibration-controlled transient elastography OR VCTE))	
CINAHL	((MH "Nonalcoholic Fatty Liver Disease" OR (metabolic	16

	dysfunction-associated steatotic liver disease OR MASLD OR nonalcoholic fatty liver disease OR NAFLD OR steatotic liver disease OR nonalcoholic steatohepatitis OR NASH)) AND (((MH "Apnea+" OR MH "Sleep Apnea, Obstructive" OR MH "Sleep Apnea Syndromes+") OR MH "Airway Obstruction+" OR MH "Polysomnography") OR (obstructive sleep apnea OR OSA OR sleep apnea syndromes OR airway obstruction OR apnea OR polysomnography OR PSG OR sleep disordered breathing OR apneic OR hypopnea OR upper airway resistance syndrome OR UARS OR Apnea Hypopnea Index OR AHI OR Epworth Sleepiness Scale OR ESS OR Oxygen desaturation index OR ODI OR Pittsburgh Sleep Quality Index OR PSQI OR Functional Outcomes of Sleep Questionnaire OR FOSQ OR Sleep efficiency OR Rosen Criteria))) AND (MH "Continuous Positive Airway Pressure" OR MH "Life Style Changes" OR (MH "Pharmacological and Biological Treatments+") OR MH "Glucagon-Like Peptide 1" OR MH "Bariatric Surgery+" OR (continuous positive airway pressure OR CPAP OR lifestyle modification OR pharmacological treatment OR pharmacological intervention OR Glucagon-like peptide-1 OR GPL-1 OR semaglutide OR Wegovy OR Ozempic OR liraglutide OR Saxenda OR Victoza OR Hypoglossal nerve stimulator OR surgical	

	intervention OR bariatric surgery OR Tirzepatide (Mounjaro) OR CagriSema OR Retatrutide OR life style changes OR drug therapy OR Pharmacological and Biological Treatments)) AND (MH "Weight Loss+" OR MH "Glycemic Control" OR MH "Blood Pressure+" OR MM "Body Composition+" OR MH "Alanine Aminotransferase" OR MH "Aspartate Aminotransferase" OR (liver fat OR liver stiffness OR circulating biomarker OR body weight loss OR metabolic disease control OR lipid change OR glycemic control OR blood pressure OR composition OR body composition OR health-related quality of life OR Alanine Transaminase OR HRQOL OR alanine aminotransferase OR ALT OR aspartate aminotransferase OR AST OR enhanced liver fibrosis OR ELF OR magnetic resonance elastography OR MRE OR magnetic resonance imaging proton density fat fraction OR MRI-PDFF OR vibration-controlled transient elastography OR VCTE))	
Web of Science	(((metabolic dysfunction-associated steatotic liver disease OR MASLD OR nonalcoholic fatty liver disease OR NAFLD OR steatotic liver disease OR nonalcoholic steatohepatitis OR NASH) AND (obstructive sleep apnea OR OSA OR sleep apnea syndromes OR airway obstruction OR apnea OR polysomnography OR PSG OR sleep disordered breathing OR	82

	apneic OR hypopnea OR upper airway resistance syndrome OR UARS OR Apnea Hypopnea Index OR AHI OR Epworth Sleepiness Scale OR ESS OR Oxygen desaturation index OR ODI OR Pittsburgh Sleep Quality Index OR PSQI OR Functional Outcomes of Sleep Questionnaire OR FOSQ OR Sleep efficiency OR Rosen Criteria)) AND (continuous positive airway pressure OR CPAP OR lifestyle modification OR pharmacological treatment OR pharmacological intervention OR Glucagon-like peptide-1 OR GPL-1 OR semaglutide OR Wegovy OR Ozempic OR liraglutide OR Saxenda OR Victoza OR Hypoglossal nerve stimulator OR surgical intervention OR bariatric surgery OR Tirzepatide (Mounjaro) OR CagriSema OR Retatrutide OR life style changes OR drug therapy OR Pharmacological and Biological Treatments)) AND (liver fat OR liver stiffness OR circulating biomarker OR body weight loss OR metabolic disease control OR lipid change OR glycemic control OR blood pressure OR composition OR body composition OR health-related quality of life OR Alanine Transaminase OR HRQOL OR alanine aminotransferase OR ALT OR aspartate aminotransferase OR AST OR enhanced liver fibrosis OR ELF OR magnetic resonance elastography OR MRE OR magnetic resonance imaging proton density fat fraction OR MRI-PDFF OR vibration-	

	controlled transient elastography OR VCTE)	
